# Aging-related anatomical and biochemical changes in lymphatic collectors impair lymph transport, fluid homeostasis, and pathogen clearance

**DOI:** 10.1111/acel.12330

**Published:** 2015-05-15

**Authors:** Valerio Zolla, Irina Tsoy Nizamutdinova, Brian Scharf, Cristina C Clement, Daisuke Maejima, Tony Akl, Takashi Nagai, Paola Luciani, Jean-Christophe Leroux, Cornelia Halin, Sabriya Stukes, Sangeeta Tiwari, Arturo Casadevall, William R Jacobs, David Entenberg, David C Zawieja, John Condeelis, David R Fooksman, Anatoliy A Gashev, Laura Santambrogio

**Affiliations:** 1 Department of Pathology, Albert Einstein College of MedicineBronx, NY, 10461, USA; 2 Department of Medical Physiology, College of Medicine, Texas A&M University Health Science CenterTemple, TX, 76501, USA; 3 Department of Physiology, Shinshu University School of MedicineMatsumoto, Japan; 4 Department of Biomedical Engineering, Texas A&M UniversityCollege Station, TX, 77843, USA; 5 Institute of Pharmaceutical Sciences, ETH ZurichVladimir-Prelog-Weg 4, Zurich, CH-8093, Switzerland; 6 Department of Microbiology and Immunology, Albert Einstein College of MedicineBronx, NY, 10461, USA; 7 Department of Anatomy and Structural Biology, Albert Einstein College of MedicineBronx, NY, 10461, USA; 8 Gruss Lipper Biophotonics Center, Albert Einstein College of MedicineBronx, NY, 10461, USA

**Keywords:** aging, mass spectrometry, oxidative stress, proteomics, lymphatics

## Abstract

The role of lymphatic vessels is to transport fluid, soluble molecules, and immune cells to the draining lymph nodes. Here, we analyze how the aging process affects the functionality of the lymphatic collectors and the dynamics of lymph flow. Ultrastructural, biochemical, and proteomic analysis indicates a loss of matrix proteins, and smooth muscle cells in aged collectors resulting in a decrease in contraction frequency, systolic lymph flow velocity, and pumping activity, as measured *in vivo* in lymphatic collectors. Functionally, this impairment also translated into a reduced ability for *in vivo* bacterial transport as determined by time-lapse microscopy. Ultrastructural and proteomic analysis also indicates a decrease in the thickness of the endothelial cell glycocalyx and loss of gap junction proteins in aged lymph collectors. Redox proteomic analysis mapped an aging-related increase in the glycation and carboxylation of lymphatic’s endothelial cell and matrix proteins. Functionally, these modifications translate into apparent hyperpermeability of the lymphatics with pathogen escaping from the collectors into the surrounding tissue and a decreased ability to control tissue fluid homeostasis. Altogether, our data provide a mechanistic analysis of how the anatomical and biochemical changes, occurring in aged lymphatic vessels, compromise lymph flow, tissue fluid homeostasis, and pathogen transport.

## Introduction

The major function of the lymphatic vessels is to transport lymph. The role of lymph transport makes lymphatics pivotal to the movement of fluid, soluble molecules, and immune cells from the interstitium of parenchymal organs to the draining lymph nodes. Networks of lymphatic vessels dispersed in interstitial spaces collect proteins and molecule ultrafiltrates from the blood capillaries, products of tissue metabolism/catabolism, tissue remodeling and injury, apoptotic cells, and pathogens and serve as a conduit for immune cell homing to the lymph nodes. Thus, altogether, the lymphatics play a major role in body fluid homeostasis, tissue proteostasis, and innate and adaptive immune responses (Dixon *et al*., 2006; Clement *et al*., 2010, 2011; Levick & Michel, 2010; Akl *et al*., 2011; Davis *et al*., 2011; Gonzalez *et al*., 2011; Platt & Randolph, 2013).

The driving force that propels the lymph forward is generated by the active spontaneous contractions of the lymphatic muscle cells that surround the basal membrane and the endothelial cells of the lymphatic vessels (Zawieja *et al*., 1993; Gashev, 2008). The activity of the vessel’s intrinsic pump is crucial to lymph transport against an opposing net hydrostatic gradient (McHale & Roddie, 1976; Zawieja *et al*., 1993; Muthuchamy *et al*., 2003; Gashev, 2008; Akl *et al*., 2011). Additionally, a set of intraluminal valves positioned along the lymphatic vessels, which close in coordination with the lymphatic muscle contractile activity, prevent lymphatic backflow at the end of each contraction (McHale & Roddie, 1976; Ohhashi *et al*., 1980; Gashev, 1991). The coordination between the pumping activity of the lymphangions, the sections of lymphatic vessels between adjacent valves (Mislin, 1961), and the closing of the valves promote the unidirectional flow of the lymph from the parenchymal tissue to the draining lymph nodes.

An important component of lymphatic transport is the presence of a thick glycocalyx on the lumen side of the endothelial cells generated by a backbone of proteoglycans and glycoproteins (Reitsma *et al*., 2007). The proteoglycans are formed by a core protein bound to glycosaminoglycan side chains (Reitsma *et al*., 2007). The core protein can be anchored to the cell membrane (syndecans, glypicans) or secreted into the glycocalyx structure (mimecan, perlecan, and biglycan). The glycosaminoglycans are bound, as side chains, to the core protein and comprise heparan, chondroitin, dermatan and keratan sulfates, and hyaluronic acid. Several more glycoproteins constitute the backbone of the glycocalyx including adhesion molecules, selectins, integrins, and Ig superfamilies, and components of the coagulation and fibrinolysis cascade. Soluble proteins, such as albumin and orosomucoid, are also retained in the glycocalyx and are pivotal, together with the proteoglycans, in maintaining its negative charge and selective permeability. The function of the glycocalyx is to aid lymphocyte rolling, to prevent immune cells from adhering to the endothelium, to generate a cytokine/chemokine gradient, and to balance the exchanges between the lymphatic and the surrounding interstitium (Reitsma *et al*., 2007; Levick & Michel, 2010).

The magnitude and the complexity of the many tasks performed by the lymphatics is highlighted by the extensive tissue edema and impaired immunity observed in mice and humans with decreased lymphangiogenesis (Swartz *et al*., 2008) or surgical removal of draining lymph nodes (Blum *et al*., 2013). Many aspects of the lymphatic transport in physiological and pathological conditions have been elucidated, but a number of important aspects of lymphatic functions have yet to be investigated. Several recent reports demonstrate that a consequence of the aging process is the loss of lymphatic muscle cells in the valvular zones of the lymphatic vessel wall (Bridenbaugh *et al*., 2013a) with signs of diminished lymphatic contractility and flow (Muthuchamy *et al*., 2003; Akl *et al*., 2011; Nagai *et al*., 2011), which in part may be linked to increased oxidative stress (Thangaswamy *et al*., 2012). Indeed, an increase in superoxide dismutase activity, lipid peroxidation, cellular superoxide, and mitochondrial ROS was reported in aged lymphatic collectors (Thangaswamy *et al*., 2012) and oxygen-derived free radicals have been shown to inhibit lymphatic contractile function (Zawieja *et al*., 1991; Zawieja & Davis, 1993).

However, the effect of aging-related changes on the movement of fluids, molecules, and cells through the lymphatic vessels, or on the lymphatic permeability remains unknown. Knowledge of how the aging process affects the dynamics of lymph flow and the function of lymphatics is important to further our understanding of the pathogenesis of altered fluid homeostasis and microvascular hyperpermeability in aging and to elucidate some of the aspects of immunosenescence and altered response to pathogens.

In this study, we provide an ultrastructural, biochemical, and functional comparative analysis between adult and aged lymphatic vessels. The observed changes provide a mechanistic explanation for the development of tissue edema and the compromised response to pathogens often observed in aging (Franceschi *et al*., 2000; Panda *et al*., 2009).

## Results

### Decreased extracellular matrix and contractile proteins in aged lymphatic collectors

**Fig 1:**
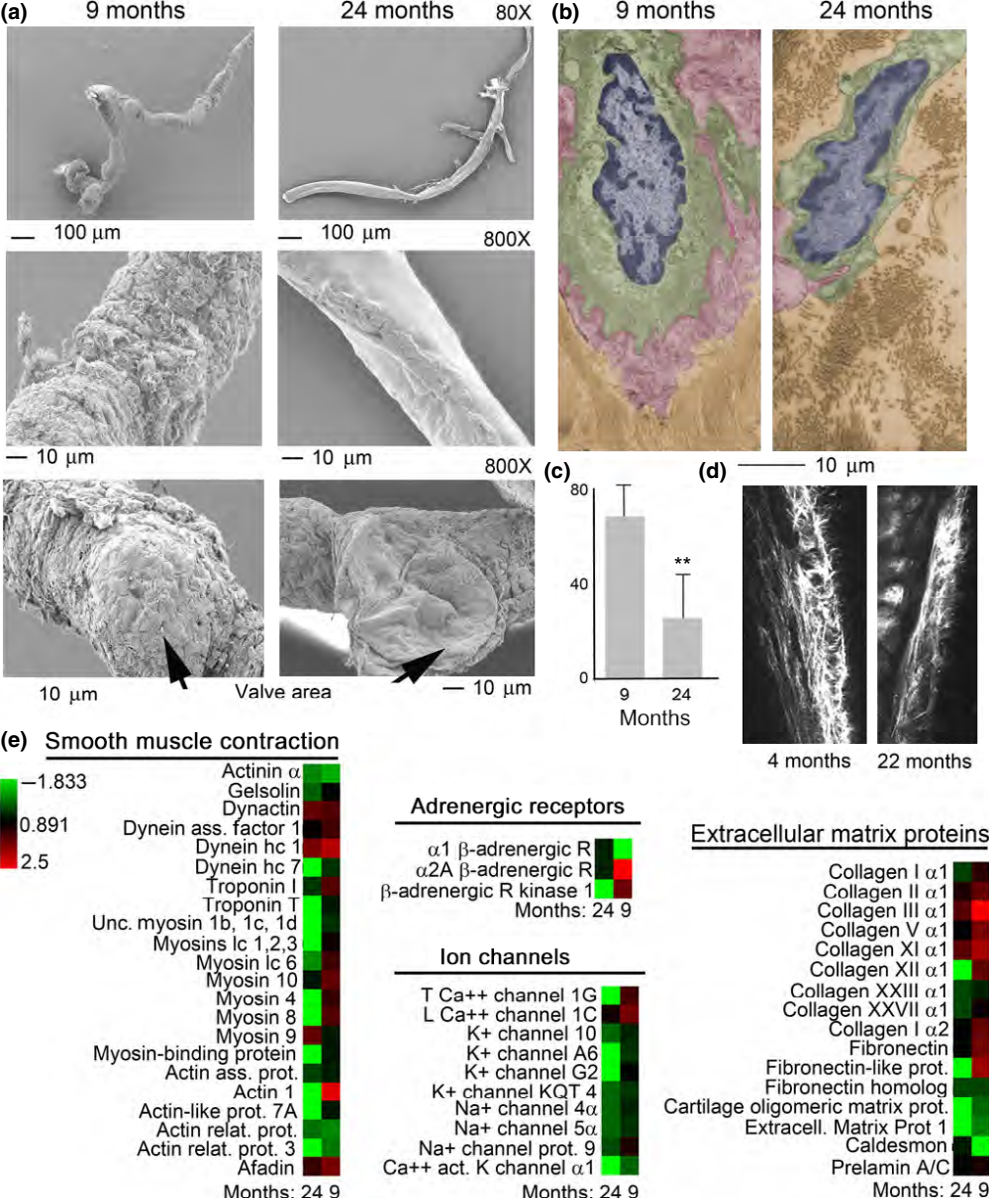
Ultrastructural and proteomic analysis of basal membrane in rat mesenteric lymphatic vessels. (a) Scanning electron micrographs of 9- and 24-month-old rat mesenteric lymphatic vessels. (b) Ultrastructural analysis of endothelial cells (nucleus colored in blue, cytoplasm colored in green, and glycocalyx colored in red) and basal membrane (colored in brown) in lymphatic collectors from 9- and 24-month-old rats. (c) Quantification of basal membrane thickness in adult versus old lymphatics. Average and standard deviation from 15 separate measurements. **P < 0.005. (d) Intravital multiphoton microscopy of collagen fibers surrounding lymphatic collector of 4- and 22-month-old mice. (e) Heat map representation of the proteomic analysis performed on 9- and 24-month-old rat mesenteric lymphatic vessels. The color-coded heat map shows proteins with the highest level of expression in red and the lowest abundance proteins in green, with intermediates shades for the rest of the expression levels. Data compile three independent biological replicates.

As a first step to determine how the aging process affects lymphatic vessels, we performed ultrastructural analysis using scanning and transmission electron microscopy (SEM and TEM respectively). Mesenteric lymphatic vessels were isolated from 9- and 24-month-old Fisher 344 rats. Scanning electron microscopic analysis of these tissues demonstrated substantial structural differences in the vessels isolated from adult and old rats (Fig.1a). Examination of the adventitia, or external surface of the 9-month-old collectors, revealed tight connective tissue surrounding the lymphatic (Fig.1a). However, lymphatic collectors isolated from 24-month-old rats exhibited tissue degeneration with a substantial loss of extracellular matrix (Fig.1a). Additionally, visual analysis of vessels from 9-month-old rats established that the collector’s valve area was surrounded by extracellular matrix, whereas a loss of extracellular matrix was observed in valves of collectors isolated from aged rats (Fig.1a).

Ultrastructural examination of lymphatic vessel thin sections by TEM also revealed a marked decrease in the extracellular matrix associated with the lymphatic collectors, with more dispersed areas of collagen bundles, surrounding endothelial cells in aged samples as compared to the adult ones (Fig.1b,c). Decreased collagen surrounding the lymphatic collectors was also observed in 22-month-old mice as compared to 4-month-old mice, by intravital two-photon laser scanning microscopy (Fig.1d).

To confirm the observed structural differences among the two age groups, we performed a global proteomic analysis on the isolated rat mesenteric lymphatic vessels. The protein expression profiling was performed on three different sample preparations each consisting of two/three isolated vessels for each age-group using one-dimensional SDS–PAGE coupled with nanoLC–ESI–MS/MS analysis of Lys-C/tryptic/Glu-C/Asp-N peptides. The three independent biological replicates for each age-group were analyzed by Scaffold analysis (version Scaffold 4.0.7, Proteome Software Inc., Portland, OR), and the label-free quantification of the protein expression in each biological sample was used to generate an unclustered heat map (Fig.1e).

Global proteomic profiling of the lymphatic vessels revealed statistically significant differences (*P* < 0.05 and a false discovery rate of <0.4) in protein expression in the two age-groups (Fig.1e and Table S1). Many muscle contractile and regulatory proteins, such as troponins, myosin, and their cytoskeleton associated proteins, such as actin, gelsolin, dynein, and myosin binding proteins were at least twofold lower in the proteome of 24-month-old lymph collectors (Fig.1e and Table S1). Other proteins, including Na^+^, K^+^, and Ca^++^ channels, known to be involved in the generation of the muscle cell action potential, induction of depolarization, and Ca^++^ release were also decreased in the aged lymphatic collectors (Fig.1e and Table S1). In addition, extracellular matrix proteins, including fibronectins, collagens, and cartilage oligomeric protein, were also substantially downregulated in the 24-month-old lymph collectors (Fig.1e and Table S1). Finally, adrenergic receptors, and associated kinase, involved in the regulation of the lymphatic vessel contractility during stress-associated events were also affected by the aging process (Fig.1e and Table S1). Altogether, the downregulation of several proteins associated with muscle contraction, generation of the action potential, Ca^++^ release, and formation of basal membrane/extracellular matrix, in large degree, parallel the differences observed in matrix and ultrastructural proteins between adult and aged lymphatic vessels (Fig.1 and Table S1).

### Decreased contractility and pumping efficiency in aged lymphatic collectors

**Fig 2:**
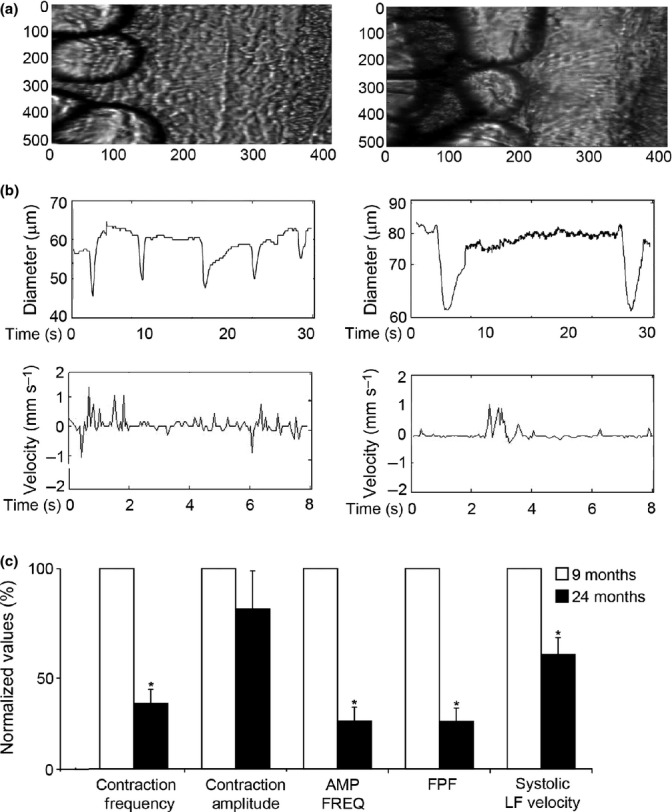
*In vivo* analysis of mesenteric lymphatic vessels contractility. (a) Microscopic view and (b) representative tracings of contractile activity of adult (9 months) and aged (24 months) rat mesenteric lymphatic vessels. (c) Aging-associated changes in vessels contractile activity. AMP*FREQ Product—amplitude–frequency product, FPF—fractional pump flow, and LF—lymph flow. Significant differences (*P* ≤ 0.05) between active lymph pump parameters indicated by * - 9-month versus 24-month specimens Movies (S1a,b).

To analyze whether aging-associated changes in lymphatic ultrastructure and proteome composition could affect the vessel functionality, we analyzed the lymphatic contractile activity of mesenteric collectors in adult and aging rats. Using intravital microscopy, we determined that, when compared to adult collectors, aged lymphatic vessels can still contract comparatively strong with around 20% decrease in contraction amplitude (Fig.2a,b and Movie S1a). However, the contraction frequency is drastically diminished; up to a 70% decrease is observed in aged collectors as compared to adult ones (Fig.2a,b and Movie S1b). These aging-associated changes in mesenteric lymphatic contractility led to a significant reduction in their pumping indices. Both the amplitude–frequency product (AFP) and the fractional pump flow (FPF) were reduced to 24% of what measured in the adult mesenteric collectors. Consequently, systolic lymph flow velocity was also significantly diminished in aged collectors to a value corresponding to almost half of what observed in the adult collectors. Altogether, the *in vivo* functional data support the notion that the aging process decreases the lymphatic collector’s functional activity.

### Decreased glycocalyx and junctional proteins in aging lymphatic collectors

**Fig 3:**
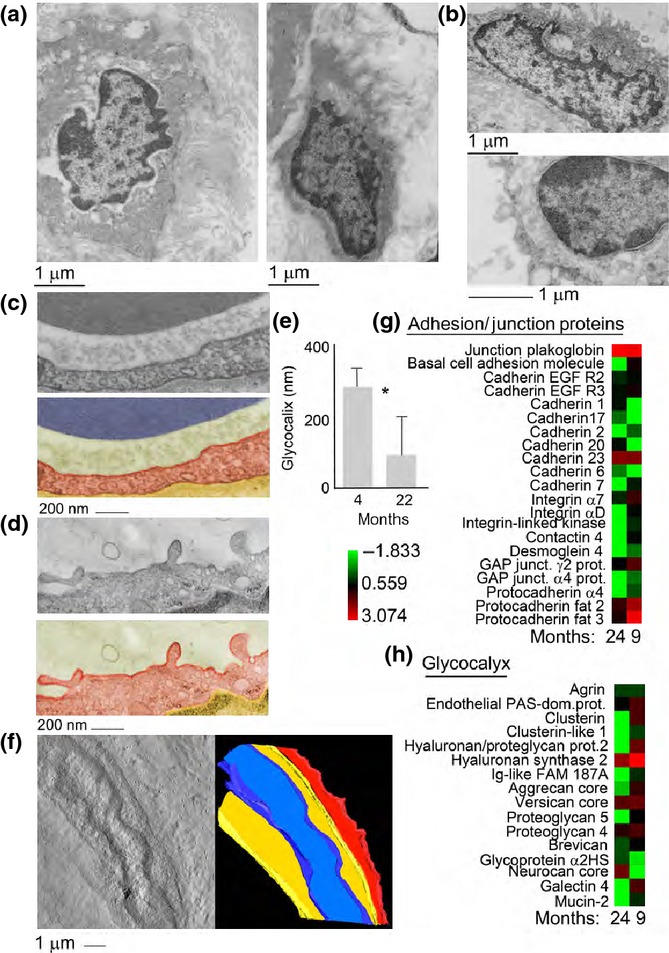
Ultrastructural and proteomic analysis of the glycocalyx in rat mesenteric lymphatic vessels. (a) Ultrastructural analysis of the lymphatic endothelial cell glycocalyx from 9-month-old mesenteric lymphatic vessels. (b) Ultrastructural analysis of the lymphatic endothelial cell glycocalyx from 24-month-old mesenteric lymphatic vessels. (c) Transmission electron micrograph and pseudocolored micrograph of the lymphatic endothelial cell glycocalyx from a 9-month-old rat 9; cytoplasm is colored in red and glycocalyx in green. (d) Transmission electron micrograph and pseudocolored micrograph of the lymphatic endothelial cell glycocalyx from a 24-month-old rat; cytoplasm is colored in red and glycocalyx in green. Bar corresponds to 200 nm. (e) Quantification of glycocalyx thickness in adult versus old lymphatics. Average and standard deviation from 15 separate measurements. *P < 0.001. (f) Tomographic reconstruction and 3D model of the lymphatic endothelial cell glycocalyx in a 9-month-old rat. Three-dimensional model view presents the glycocalyx coat in red, the cytosol in yellow, and the nucleus in blue. Bar corresponds to 1 μm. (g and h) Heat map representation of the proteomic analysis performed on 9- and 24-month-old rat mesenteric lymphatic vessels. The color-coded heat map shows proteins with the highest level of expression in red and the lowest abundance proteins in green, with intermediates shades for the rest of the expression levels. Data compile three independent biological replicates.

The glycocalyx is an important component of the endothelial barrier in lymphatic collectors. To examine possible age-related ultrastructural changes to the endothelial glycocalyx, lymphatic collectors were isolated from 9- and 24-month-old rats, stained with Alcian blue and examined by transmission electron microscopy. The glycocalyx of endothelial cells from 9-month-old rats was observed as an intact, continuous, electron-dense material covering the cell membrane (Fig.3a,c). On the other hand, a significant loss of the glycocalyx, with reduction in size and continuity, was observed in lymphatic endothelial cells from 24-month-old rats (Fig.3b,d,e). Electron tomography and 3D modeling were employed to examine the glycocalyx of Alcian blue-stained endothelial cells from 9-month-old rats. The tomographic analysis confirmed a well-demarcated glycocalyx of varying height spanning the luminal side of an endothelial cell in collectors from 9-month-old rats (Fig.3f).

Possible proteomic differences among glycocalyx proteins from 9- and 24-month-old lymphatic collectors were also explored. Glycoproteins were separated from the collector’s total proteome and run on a SDS–PAGE followed by in-gel digestion and MS/MS analysis as described above.

Global proteomic profiling of the lymphatic vessels revealed statistically significant differences (*P* < 0.05 and a false discovery rate of <0.4) in proteins expression between the two age-groups (Fig.3g,h and Table S1). Cadherins, protocadherins, integrins, and GAP junction proteins all showed a significant decrease (at least twofold, *P* < 0.05) in collectors harvested from aging as compared to adult rats (Fig.3g,h and Table S1). Additionally, complementing the ultrastructural analysis on the glycocalyx, proteomic analysis determined that several glycocalyx-associated proteins, including versicans, aggrecans, proteoglycans, brevican, galectins, mucins, agrin, and clusterins were at least twofold downregulated in aged collectors as compared to adult ones (Fig.3g,h and Table S1). Altogether, the downregulation of several proteins associated with glycocalyx and GAP junction supports the aging-related changes observed by ultrastructural microscopy.

### Increased oxidative stress in aged lymphatic collectors

**Fig 4:**
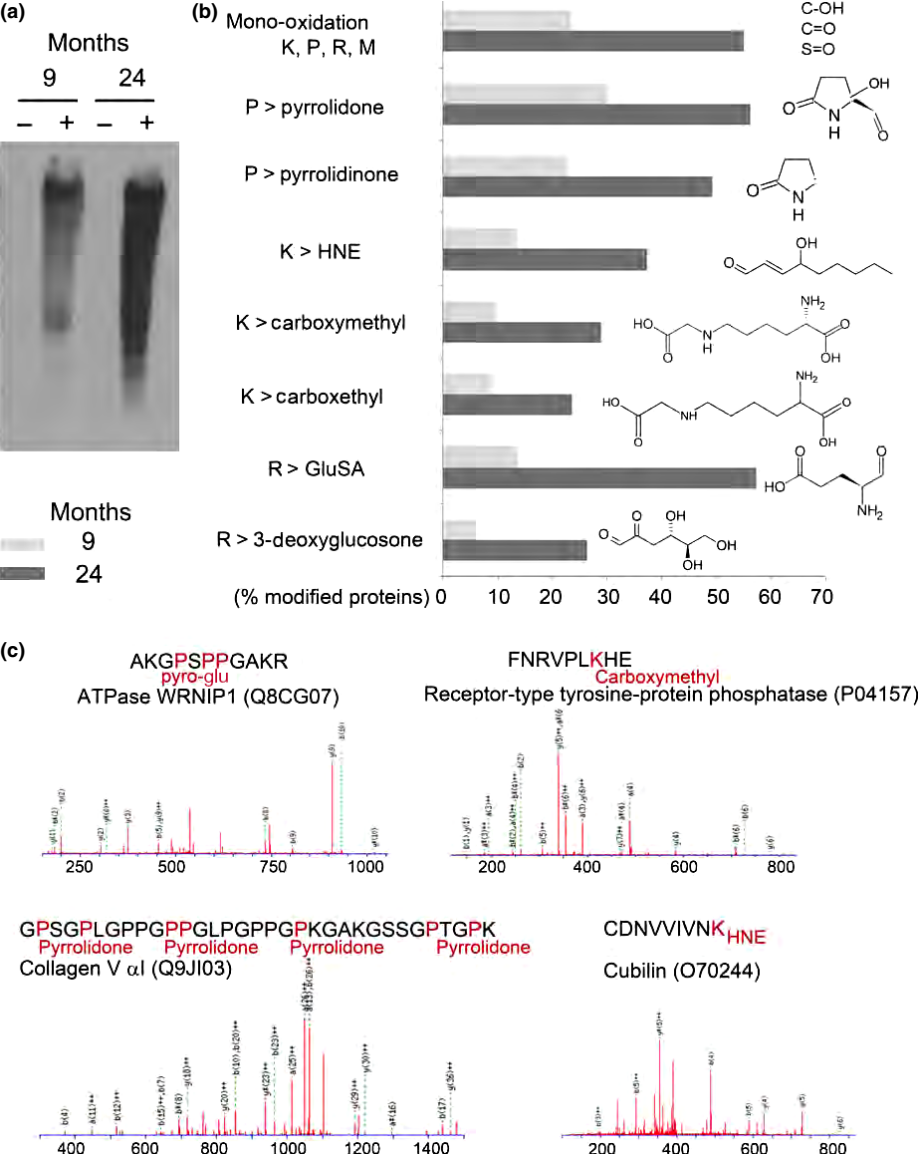
Increased oxidative stress and protein carbonylation in aged MLV. (a) Western blot analysis of oxidatively modified (carbonylated) proteins detected in MLV isolated form 9- and 24-month-old rats. Lanes marked as ‘-’ indicate nonderivatized proteins (control) and ‘+’ indicate derivatized proteins. (b) Number (expressed in %) of proteins with posttranslation oxidative modifications. (c) Examples of MS/MS mapping of oxidative modifications of amino acid side chains across the peptide sequences in MLV proteomes isolated from 24-month-old rat shows Pyro-Glu, carboxymethyl, pyrrolidone, and HNE modifications in ATPase WRNIP1, receptor-type tyrosine protein phosphatase, collagen Vα1, and cubilin, respectively. Data compile three independent biological replicates.

Aging-related increases in oxidative stress have been associated with endothelial dysfunction and a diminution of mesenteric lymphatic vessel contractility (Thangaswamy *et al*., 2012). To examine whether an aging-associated increase in oxidative stress would result in an elevation of the oxidatively modified proteins comprising the mesenteric lymphatic vessel, proteins were extracted from 9- and 24-month-old rat lymphatic collectors and incubated with 2,4-dinitrophenylhydrazine (DNPH). DNPH specifically reacts with carbonylated proline, lysine, histidine, and arginine residues. Derivatized samples were separated by gradient SDS–PAGE and analyzed by Western blotting using an anti-DNPH primary antibody. Protein carbonyls were detected in mesenteric lymphatic vessels isolated from 9- and 24-month-old rats (Fig.4a). However, the accumulation of carbonylated proteins was significantly elevated in the lymphatic collectors from 24-month-old rats (Fig.4a). To further probe into lymphatic endothelial protein oxidative damage, we isolated mesenteric lymphatic vessels from 9- and 24-month-old rats and their extracted proteins were run on an SDS–PAGE and analyzed by one-dimensional liquid chromatography coupled with tandem mass spectrometry on a nanoLC/Orbitrap system. In 24-month-old collectors, the percentage of chemically modified proteins was significantly higher as compared to the adult samples (Fig.4b). The most frequent posttranslational modifications (PTM) we identified were as follows: mono-oxidation, pyrrolidinone, 4-hydroxynonenal (HNE), carboxymethyl, carboxethyl, glutamic semialdehyde (Glu SA), and 3-deoxyglucosone. Mono-oxidation on lysine, proline, methionine, and arginine was found to be about 55%, while the pyrrolidone and pyrrolidinone derivatives of proline were present in higher abundance (56.2 and 49.2%, respectively). Arginine conversion to GluSa (57.3%) and 3-deoxyglucosone (26.3%) was another posttranslational oxidative modification that was significantly increased. In addition, the lysine residues were observed to be significantly modified as four Hydroxynonenal (37.3%), carboxymethyl (29%), and carboxethyl (23.6%). In the 9-month-old samples, the percentages of previously stated modifications were generally lower (Fig.4b,c). Altogether, the data support the presence of increased oxidative stress in aged lymphatic collectors.

### Impaired pathogen clearance by aged lymphatic collectors

Reduced innate and adaptive immune responses are a hallmark of aging. Several reports have indicated how aging antigen-presenting cells have a decreased ability to phagocytose and process pathogens and aging T cells have decreased proliferative responses (Franceschi *et al*., 2000; Panda *et al*., 2009). However, so far, little is known about how the aging process affects bacterial clearance associated with lymphatic transport.

**Fig 5:**
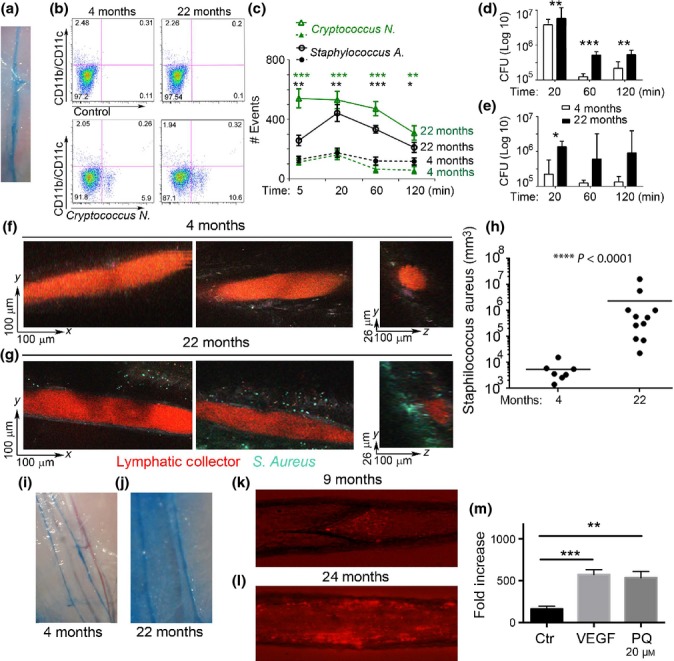
Compromised pathogen transport and increased lymphatic permeability in aging mice. (a) Mice lymphatic collector labeled with Evans Blue. (b) FACS analysis of *Cryptococcus neoformans* presents in lymphatic collectors, as free bacteria or phagocytosed by CD11c^+^/CD11b^+^ cells. (c) Quantification of *Cryptococcus neoformans* and *S. aureus,* present at different time points, in the lymphatic collectors following injection in the hind limb footpad of 4- and 22-month-old mice. *P < 0.05, **P < 0.01; ***P < 0.001; ****P < 0.0001. (d) Amount of *Cryptococcus neoformans* and (e) *Mycobacterium smegmatis*, as measured by colony-forming unit, present in the same sample as in (c). (f–g) Representative (xy and yz) images of lymphatic collectors in the hind leg taken 1 h after footpad injection with a TRITC–dextran sinus marker (red) and *S. Aureus* (cyan) bacteria in the footpad from 4-month-old mice (f) and 22-month-old mice (g). (h) Density of bacteria in the surrounding footpad was quantified in both groups and compared by Mann–Whitney test in four or more mice per condition, taken from three experiments. (i and j) Evans blue distribution in the calf lymphatic collectors after lower limb footpad injection in 4-month-old mice and (j) 22-month-old mice. Representative image from four independent experiments k and l) Representative images of 9-month-old rats (k) and 24-month-old rats (l) isolated and cannulated segments of mesenteric lymphatic vessels under conditions of perfusion of bacteria-containing solution. (m) Quantification of FITC–dextran molecules passing through a monolayer of lymphatic endothelial grown to confluence on Transwell filters. A FITC–dextran (70 kDa) solution was added to the upper chambers, and the concentration of fluorescence (i.e. absorbance) in the lower chamber was measured 15 min later using a spectrophotometer. VEGF-A (20 nm) as well as paraquat (20 μM) significantly enhanced LEC permeability over control levels. Data from one representative out of three similar experiments are shown. **P < 0.01; ***P < 0.001; ****P < 0.0001.

To study whether aging-related anatomical, biochemical, and functional differences would interfere with pathogens clearance, we set up different functional assays using three different pathogens, *Staphylococcus aureus*, *Cryptococcus neoformans*, and *Mycobacterium smegmatis*. Pathogens (1 × 10^8^ − 1 × 10^9^ CFU) were injected in the footpad of 4- and 22-month-old mice, and draining lymphatics from the lower limbs, below the popliteal node, were collected at different time points (5 min, 20 min, 1 h, and 2 h) (Fig.5a). Following tissue digestion with collagenase, free fluorescently labeled pathogens transported by the lymphatic collectors were analyzed by flow cytometry and quantified (Fig.5b). Over the analyzed time course, more than 85% of the pathogens were present as free bacteria or fungi (Fig.5b). The total number of free pathogens was consistently and significantly higher in samples collected from aged mice, at each of the examined time points (Fig.5c). *C. neoformans* and *M. smegmatis* were also quantified by colony-forming units; again, the number of CFU was significantly higher in aging samples (Fig.5d).

To further analyze pathogen transport in the lymphatic collectors, we performed intravital two-photon microscopy of lymph-carried pathogens (Movies S2a,b,c). GFP-expressing *S. aureus* (1 × 10^8^ CFU) and a fluorescent tracer, (tetramethylrhodamine isothiocyanate (TRITC)–dextran or PEG-D680) (Proulx *et al*., 2013) (Movie S3), were injected in the lower limb footpads of 4 and 22-month-old mice to visualize the transit of the bacteria through the collectors in the legs. In both groups, we could visualize transit of the bacteria through the collectors in the leg (Movies S2a,b,c). In separate experiments, mice were injected with bacteria 1 h prior to surgery, to allow transport through the collectors, and subsequently imaged. In both groups of mice, bacteria could be sporadically found within the vessel space (Fig.5f,g). In young mice, there were occasionally bacteria found in the fat pad surrounding the collectors; however, in aged mice, we observed 100 times more bacteria accumulated in the fat pad and along the outside of the vessel (Fig.5h). These bacteria-rich regions were unevenly distributed within the fat, suggesting that the lymphatic leakage from the vessels into the surrounding interstitium was more predominant in selective areas of the lymphangions.

### Impaired permeability in aged lymphatic collectors

Because time-lapse microscopy clearly indicated that bacteria could easily exit from aged lymphatic collectors and because structural and proteomic differences were observed in the aging glycocalyx, we set out to further analyze the lymphatic collector’s permeability. To this end, Evans blue dye was injected in the footpad of hind limb of 4- and 22-month-old mice. After 20 min, mice were perfused *via* the left ventricle with PBS, and the collecting lymphatic vessels of mice were imaged under intravital microscopy. In young mice, the dye was mostly confined to the collectors (Fig.5i), while in the aged mice, the Evans blue was present both in the collectors and in the surrounding tissue (Fig.5j).

Similar results were obtained on isolated, cannulated, and pressurized rat mesenteric lymphatic vessels under transmural pressure of 5 cm H_2_O. Bacteria were injected to the tubing connected to the input end of the collector and their transport visualized by epifluorescent microscopy (Fig.5k,l). In adult collectors, the bacteria were observed moving along the vessel and often can be visible close to the lymphatic valve, before its opening which propelled them downstream (Fig.5k). On the other hand, in aged collectors, the bacteria were mostly seen lingering close to the vessel wall (Fig.5l) and often visually observed exiting the lumen through the lymphatic wall.

To recapitulate the experiment on aging collectors, a Transwell plate assay was performed to assess permeability of human lymphatic endothelial cells, after treatment with paraquat, which is known to induce aging-like oxidative stress. Lymphatic endothelial cells were grown to confluence on Transwell filters, and FITC–dextran was added to the upper Transwell chambers. The integrity of the endothelial cells monolayer was subsequently determined spectrophotometrically by measuring the concentration of FITC–dextran that had diffused into the bottom well over 15 min. In this setup, overnight treatment with paraquat (20 μM) significantly increased lymphatic endothelial cells permeability (Fig.5m). Notably, this effect was comparable to the one induced by 20 min of treatment with vascular endothelial growth factor (VEGF)-A (20 nM), a well-known inducer of vascular permeability (Fig.5m) (Ferrara *et al*., 2003).

## Discussion

The lymphatic system is involved in several functions such as tissue fluid homeostasis and innate and adaptive immunity. It comprises blunt-ended lymphatic capillaries, collecting lymphatic vessels, lymph nodes, and the thoracic duct.

The lymph capillaries begin in the interstitial space of all parenchymal organs, with the exception of the retina, bone, and brain, and collect tissue fluids, cells, and products of tissue metabolism/catabolism and cellular secretion. The capillaries converge into precollectors and collecting lymphatic vessels, which directly drain into the lymph nodes, merge, and eventually transport the lymph into either the thoracic duct or the right lymphatic duct that joins the subclavian veins. Thus, ultimately, the lymphatic system converges with the blood circulation.

In the capillaries, the lymphatic endothelial cells are both supported by a thin basement membrane which lacks pericytes and the endothelial cells are directly anchored to the extracellular matrix through filament bundles (Skalak *et al*., 1984; Mazzoni *et al*., 1987; Pflicke & Sixt, 2009; Tammela & Alitalo, 2010). This allows the organ’s interstitial pressure to control the fluid flux into the lymph capillaries; when the interstitial pressure increases, the filaments open the endothelial cells junctions to facilitate fluid entry into the capillaries (Leak & Burke, 1966). Alternatively, the endothelial cells in the lymph collectors have tight junctions, as they are specialized in lymph transport instead of formation; thus, they are supported by a basal membrane and also surrounded by lymphatic muscle cells whose contractions propel the lymph fluid forward (McHale & Roddie, 1976; Ohhashi *et al*., 1980). Additionally, several bileaflet valves are present throughout the length of the collectors, which by closing in unison with each contraction, minimizing retrograde lymph flow (Benoit *et al*., 1989; Zawieja *et al*., 1993; Davis *et al*., 2011).

Our current analysis mapped several anatomical and functional changes that are observed in the aged lymphatic collectors when compared to the adults. The ultrastructural and proteomic analysis indicated a loss of the basal membrane and the extracellular matrix supporting the lymphatic endothelial cells as well as the proteins related to GAP junction formation. Functionally, the aged lymphatic vessels were impaired in their ability to actively support lymph flow. Significant reduction in pumping indices, including amplitude, frequency, and fractional pump flow, were observed. Under resting conditions these changes can generate low level of tissue edema, particularly when associated with increased vessel permeability as also observed in this study. However, in pathological conditions, such as acute and chronic inflammation, the increased volumetric loads imposed on the lymphatic collectors could further enhance their impaired ability to support the lymph flow.

A reduced thickness in the glycocalyx, with increased protein glycation and oxidation, was also observed. These modifications help explain the increased permeability observed in the aged collectors that results in increased extravasation of bacteria and fungi and a decreased ability to maintain fluid transport. Microvascular dysfunction with hyperpermeability was also previously observed in aged blood vessels (Oakley & Tharakan, 2014) and was attributed to oxidative stress, inflammation, and activation of apoptotic signaling (Csiszar *et al*., 2003; Brandes *et al*., 2005; Krouwer *et al*., 2012). Likely, the same mechanism contributes to the hyperpermeability observed in the aged lymphatic collectors. Indeed, Western blot and proteomic analysis confirmed the presence of posttranslational modifications associated with oxidative stress. These modifications can alter proteins half-life, increase protein degradation, and decrease cellular functionality (Scharf *et al*., 2013). Indeed, several of the collagen proteins as well as cadherins and GAP junction proteins were decreased in aging collectors. Disruption of these proteins was previously observed to be associated with paracellular permeability in blood capillaries (Michel, 2004; Childs *et al*., 2007; Levick & Michel, 2010; Tharakan *et al*., 2012). Similarly, we observed endothelial cells barrier dysfunction and increased permeability in aged lymphatic collectors as assessed by Evans blue injection. Functionally, the ability of pathogens to more readily escape the aged lymphatic collectors would contribute to the decreased ability of the immune system to control infections in aging. Indeed, decreased lymph transport to the lymph nodes is associated with an increase in the number of tissue colony-forming units.

The lymphatic system is also the major pathway for immune cell trafficking from parenchymal organs to the lymph nodes (Randolph *et al*., 1999). Under chemokine and cytokine gradients, monocytes, dendritic cells, T cells, and B cells are transported to the draining lymph nodes (Swartz *et al*., 2008; Gonzalez *et al*., 2011; Haessler *et al*., 2011; Bridenbaugh *et al*., 2013b; Platt & Randolph, 2013). Even though we did not directly address cell trafficking in our analysis, the quantitative and qualitative changes in the glycocalyx composition and permeability of aged lymph collectors are likely to have an effect on immune cells homing to the lymph nodes (Randolph *et al*., 1999; Csiszar *et al*., 2003; Haessler *et al*., 2011; Blum *et al*., 2013; Bridenbaugh *et al*., 2013b) in elderly.

Altogether, our analysis mapped the complexity of the aging-related anatomical, biochemical, and functional changes in lymphatic collectors. The decreased ability to transport bacteria to the draining nodes, associated with increased permeability and bacterial escape in the surrounding tissue, can contribute to the decreased ability of the immune system to clear pathogens in the elderly, as observed in immunosenescence. Aging-associated lymphatic vessel hyperpermeability, due to a decreased glycocalyx and GAP junctions, could also affect the transport of large molecules, lipids, proteins, and products of tissue metabolism/catabolism (Ahn & Simpson, 2007; Meng & Veenstra, 2007; Clement *et al*., 2010, 2011; Fang *et al*., 2010; Clement & Santambrogio, 2013), besides impairing fluid homeostasis as observed here.

## Materials and methods

### Rats and procedures for *in vivo* measurements of lymphatic contractility

For the current studies, we used Fischer 344 (F-344) male rats (animals obtained from aged rats colony maintained by NIH National Institute of Aging). The animals represented two age-groups: adult and aged (9 and 24 months old). All animal procedures for the current studies were reviewed and approved by the Institutional Animal Care and Use Committees and were in accordance with federal and local regulations.

To visualize mesenteric lymphatic vessels, rats were anesthetized with a solution containing a combination of fentanyl/droperidol (0.3 mL kg^−1^ IM) and diazepam (2.5 mg kg^−1^ IM). A 4-cm-long midline abdominal incision was made through the skin, underlying fascia, and muscle layers. A small loop of intestine, 6–7 cm in length, was exteriorized through the incision. A section of the mesentery containing lymphatic vessels was positioned and secured in the observation chamber within the field of view of the intravital microscope. Throughout the duration of the experiment, the animal was located on a heated board; its heart rate and arterial blood oxygenation were monitored using a Nonin IPX1 pulse oximeter (Nonin Medical Inc., Plymouth, MN, USA). The exteriorized part of mesentery was constantly suffused (flow exchange rate 0.6 mL min^−1^) with prewarmed 38 °C albumin–physiological salt solution (APSS) (in mm: 145.0 NaCl, 4.7 KCl, 2.0 CaCl_2_, 1.2 MgSO_4_, 1.2 NaH_2_PO_4_, 5.0 dextrose, 2.0 sodium pyruvate, 0.02 EDTA, 3.0 MOPS, and 10 g L^−1^ bovine serum albumin) with pH adjusted to 7.36 at 38 °C. Mesenteric lymphatic vessels suitable for observation (i.e., located clear of adipose tissue to allow monitoring of the lymphatic diameter changes and white blood cell flow within vessels) were identified and monitored as described below. After the completion of the experiment (as well as after isolating of segments of the mesenteric lymphatic vessels, described in section below), the rat was euthanized with pentobarbital (120 mg kg^−1^ body weight IP) and verified by thoracotomy.

The average body weight of the rats used in this study was 422 ± 12 g in 9-month-old rats and 416 ± 23 g in 24-month-old rats. It should be noted that for the F-344 NIH NIA rat strain, the majority of the weight gain occurs before 7 months of age (Turturro *et al*., 1999). Therefore, both 9- and 24-month-old age-groups are within ranges of weight slightly above 400 g and are not significantly different.

### *In vivo* analysis of lymphatic contractility

In animals of both ages, the data collection was performed under conditions, while the mesenteric segment of interest was suffused only by APSS. One or two data sets (duration of 1–2 min each) were recorded within 10 min after achieving stable contractility of the observed lymphatic vessel. If more than one data set was recorded for control conditions, these data sets were averaged within one experimental day and presented in the Results as *n* = 1.

The preparation board was placed on the stage of an intravital microscope equipped with a high-speed camera (Phantom V5.2, Vision Research, Wayne, NJ, USA) triggered using a signal generated by a data acquisition board (PCI 6010, National Instruments, Austin, TX, USA). To measure the diameter of the lymphatic vessel under investigation, the vessel walls were tracked throughout the recording using a custom-designed automated, correlation-based algorithm similar to that described in details in (Dixon *et al*., 2006; Akl *et al*., 2011; Davis *et al*., 2011). We used the continuous diameter tracings to define systole and diastole in reference to the lymphatic contractile cycle (Zawieja *et al*., 1991; Gashev *et al*., 2004). The end-diastolic and end-systolic points in the diameter tracings were recorded. From the lymphatic end-diastolic and end-systolic diameters, the following lymph pump parameters were calculated:

1. Contraction frequency (FREQ), the number of contractions per minute.
2. Normalized contraction amplitude (nAMP), the difference between the end-diastolic diameter (EDD) and end-systolic diameters (ESD) normalized to end-diastolic diameter, nAMP = (EDD − ESD)/EDD.
3. Amplitude–frequency product (AFP), AFP = nAMP × FREQ, was calculated as an index of minute pumping (Davis *et al*., 2008).
4. Fractional pump flow (FPF), another index of minute lymph pump flow (minute pumping), FPF = EF × FREQ, where ejection fraction (EF) is the fraction of end-diastolic volume ejected during a single phasic lymphatic contraction, EF = (EDD^2^ − ESD^2^)/EDD^2^.

Parameters of the mesenteric lymphatic vessels contractility were normalized to those observed in adult animals to compare the degree of their aging-associated changes as previously reported (Akl *et al*., 2011).

Statistical differences were determined by two-way ANOVA, regression analysis, and paired Student’s *t*-test (JMP software version 9.0.0. for Windows) as appropriate and considered significant at *P* < 0.05. Only one lymphatic vessel was monitored in each animal.

### Mice and *in vivo* procedure

C57BL/6J mice (4 and 22 months old) were purchased from Harlan NIA as part of the age-controlled NIH mouse colony program. All animal procedures were carried out according to a protocol approved by the Institutional Animal Care of Albert Einstein College of Medicine. In some experiments, mice were injected in the footpad of both legs with tree different pathogens: a nonvirulent strain of *C. neoformans* (H99), stained with 1% Uvitex 2B, with *M. smegmatis* expressing GFP, and with *S. aureus* (Wood strain without protein A) BioParticles®, Alexa Fluor® 488 conjugate (Molecular Probes®, San Diego, CA, USA).

An amount between 1 × 10^7^ was injected in each leg with a 3/10 mL BD Lo-Dose™ (Franklin Lakes, NJ, USA) U-100 insulin syringe with 29 G × 1/2 in. BD Ultra-Fine™ (Franklin Lakes, NJ, USA) IV permanently attached needle. After different time points (5 min, 20 min, 1 h, and 2 h), the popliteal lymph node (PLN) and collector running from the injection site to the PLN were collected. The derma of the injection site was collected after 5 min as well.

### Growth conditions and CFU determination for *Cryptococcus neoformans* H99 (Serotype A)

Individual colonies of *C. neoformans* were cultured on Sabouraud dextrose agar (Difco) plates. A single colony was selected and inoculated in 10 mL of Sabouraud dextrose broth (Difco) on a shaker for 24 h at 30 °C at a speed of 250 rpms. This culture was frozen down in 10% freezing medium consisting of 10% dimethylsulfoxide (DMSO) and 90% fetal calf serum. Two days prior to the experiment, a portion of this stock was used to inoculate 10 mL of Sabouraud dextrose broth at the speed and time mentioned above for a period of 48 h. On the day of the experiment, 1 mL of the culture was pelleted and washed three times with sterile PBS before further experimental use. Samples were diluted and plated (2 plates/dilution) on Sabouraud dextrose agar plates which were then incubated for a period of 48 h at 37 °C to determine colony unit formation.

### Growth conditions and CFU determination for *Mycobacterium smegmatis* (mc^2^155)

A single colony of mc^2^155 was inoculated in liquid Middlebrook 7H9 (Difco) medium at 37 °C. GFP-expressing *M. smegmatis* was prepared by electroporation of pYUB 1588 plasmid (pMV261 expressing GFP under hsp60 promoter) into *M. smegmatis* electrocompetent cells prepared as previously described (Sweeney *et al*., 2011). Colonies were selected on solid Middlebrook 7H10 medium (Difco) plates supplemented with 10% OADC, 0.2% glycerol, and 0.05% Tween 80 (Sigma) and kanamycin (20** **μg mL^−1^). Plates were incubated at 37 °C in sealed foil. A single colony was selected for subsequent experiments. For CFU determination, samples were diluted and plated (two plates/dilution) on solid Middlebrook 7H10 medium plates, prepared as described above. Plates were then incubated for a period of 72 h at 37 °C.

### Transmission electron microscopy and electron tomography

Rat lymphatic collectors were fixed in 2.5% glutaraldehyde and 2% paraformaldehyde, in sodium cacodylate buffer 0.1 m, pH 7.4, for 3 h at 4 °C. Samples were incubated in 0.05% Alcian blue 8GX (Sigma-Aldrich, St. Louis, MO) in 0.1 m sodium cacodylate for 1 h followed by three washes with 0.1 m sodium cacodylate. Samples were postfixed in 1% osmium tetroxide and 1% lanthanum nitrate in 0.1 m cacodylate for 2 h at room temperature. Tissues were subsequently dehydrated in a series of water/ethanol mixtures to 100% and infiltrated in sequentially increasing concentrations of LX112–Araldite (Ted Pella Inc., Redding, CA, USA). Ultrathin sections were stained with uranyl acetate followed by lead citrate and viewed with a Jeol JEM-1200EX transmission electron microscope (Jeol Ltd, Peabody, MA, USA) at 80 kV.

Tomographic analysis was performed on 200-nm sections labeled with fiducial gold (10 nm) applied to both sides of the sections. Tilt series were recorded using a Tecnai 20 FEI electron microscope with a dual tilt holder at 200 kV. Stack files were collected with the region of interest tilted in increments of 1.5° within a range of −60° to +60° at 11 500×. Tilt series were combined to form a single tomogram using the IMOD tomographic and 3D reconstruction software (Kremer *et al*., 1996; Mastronarde, 1997). Contouring models of the glycocalyx and membranes were generated through IMOD guided tracking of prominent contrasted lines and planes in the tomograms.

### Scanning electron microscopy

Isolated rat lymphatic collectors were immediately fixed in 2.5% glutaraldehyde in 0.1 m sodium cacodylate buffer, pH 7.4, for 3 h at 4 °C. Tissues were dehydrated through a graded series of ethanol and critical point dried using liquid carbon dioxide in a Tousimis Samdri 790 Critical Point Drier. Dried samples were mounted on stubs and sputter-coated with chromium in an EMS150T-ES coating unit (Electron Microscopy Sciences, Hatfield, PA, USA). Samples were examined in a Zeiss Supra 40 field-emission scanning electron microscope using an accelerating voltage of 3 kV.

### Western blot analysis of oxidized proteins

Rat lymphatic collector homogenates were derivatized using the Oxyblot Protein Oxidation Detection Kit (Millipore, Billerica, MA, USA). Samples were separated on a 4–15% gradient SDS–PAGE, and transferred membranes were incubated with a rabbit polyclonal anti-DNP antibody (Millipore) and subsequently with a goat anti-rabbit Ig-HRP antibody. Carbonylated proteins were visualized by chemiluminescence, and densitometric values were calculated using Image J Software (NIH).

### Preparation, imaging, and analysis of bacterial transport in the lymphatic collectors

For imaging lymphatic collectors using the PEG-D680 (REF) dye, a custom-built two-laser multiphoton microscope (Entenberg *et al*., 2011) based on an Olympus IX-71 stand was utilized. Imaging was performed at both 880 nm from the femtosecond pulsed laser (Tsunami, Spectra Physics, Santa Clara, CA, USA) and 1260 nm from the optical parametric oscillator (Opal, Spectra Physics) to excite the PEG-D680 dye as well as to generate the SHG signal from the surrounding collagen. Five microlitre of PEG-D680 (Proulx *et al*., 2013) (50 μm) was injected in the footpad (Movie S3) immediately prior to imaging. Mice were anesthetized with ketamine (80 mg kg^−1^) and xylazine (10 mg kg^−1^), and the hind limbs were shaved. The skin along the midline dorsal side of the leg, adjacent to the lateral marginal vein, between calf and thigh was exposed. To better visualize flux cells through the collectors for time-lapse imaging, dissection of the fat covering the collectors was performed under a stereoscopic dissecting scope. Four-dimensional imaging was performed to a depth of 70 μm with images taken every 5 μm and every 1.2 min for up to 4 h. Vital signs were continuously monitored via a pulse oximeter (MouseOx, Starr Lifesciences, Oakmont, PA, USA), and physiological temperatures were maintained with a forced heated-air environmental control chamber.

For imaging bacterial transport through the lymphatic collectors, young and aged mice were injected with 1 × 10^8^ CFU of *S. aureus* expressing GFP under the control of the endogenous Agr promoter (a gift from Jan Liese) (Liese *et al*., 2013). To visualize the lymphatic, 66 kD dextran (5 μg, Sigma) conjugated in-house to trimethylrhodamine (TRITC) was synthesized and purified as described previously (Proulx *et al*., 2013) by conjugating a 20 kDa methoxypoly (ethylene glycol) amine (Sigma) to IRDye® 680LT NHS ester (LICOR Biosciences, Lincoln, NE, USA).

For the analysis of the distribution of bacteria, *S. aureus* was delivered 1 h prior to surgery, while the dextran was injected immediately just prior to surgery. Mice were euthanized, and minimal dissection of connective tissue was performed for distribution analysis.

For time-lapse imaging, both bacteria and dextran were injected together immediately prior to surgery and imaging which followed similar procedures to those described above. Images were collected on an Olympus FV-1000MPE upright laser scanning microscope with 25 × 1.05NA water immersion objective using a Spectra physics DeepSee-MaiTai Ti: sapphire pulsed laser for excitation, with emission filters for the detection of second harmonic/collagen, GFP, and rhodamine. For time-lapse movies, a single *z* position through the center of the vessel was used and collection was taken at the highest frame rate. For the analysis of the distribution of bacteria, approximately 400 × 400 × 100 μm volumes were collected with resolution of 0.8 micrometers in *x* and *y* and 5 μm in *z*.

Images were analyzed on Volocity 6.3 (Improvision). The frequency of bacteria around the collectors was measured by automatic detection of total volume of bacteria (GFP^+^), divided by the volume of a single bacterium, and normalized to the total volume of the field measured. Similar volumes were collected for all fields. This measurement was taken in multiple fields for both groups of mice, in three or more mice per condition, from three independent experiments. Means were compared by Mann–Whitney test using Graphpad 6.0 (Prism).

### Evaluations of bacterial traffic through isolated mesenteric lymphatic vessels

Once exteriorized, the isolated segments of rats MLVs were transferred to an isolated vessel chamber (modified Living Systems Instrumentation single vessel chamber model CH/1) filled with prewarmed to 38 °C D-MEM/F12 solution (Invitrogen Corp., Grand Island, NY, USA), and isolated MLV segments were cannulated and tied onto two carefully matched glass micropipettes (Gashev *et al*., 2004). The inflow and outflow pipettes were connected to independently adjustable pressure reservoirs filled with D-MEM/F12 solution. Care was taken to ensure that there were no air bubbles in the tubing or the pipettes. Once the segments of MLVs were cannulated, a slight positive transmural pressure 3 cm H_2_O was applied to detect leaks and to ensure that the vessels were undamaged and untwisted. The vessels were set to their approximate *in situ* length and positioned just above the glass coverslip comprising the chamber bottom. The chamber was transferred to the stage of an Olympus CKX41 fluorescent microscope equipped with a Lumen 200 Fluorescence Illumination System and various filters. The vessels were set to an equilibration transmural pressure of 3 cm H_2_O and slight imposed flow gradient of 1 cm H_2_O at 38 °C for 30 min. Once tone and spontaneous contractions were observed, the vessels were allowed to equilibrate for another 15 min to perform imaging in control conditions. During all experiments, segments of MLVs were constantly superfused and perfused with prewarmed 38 °C D-MEM/F12, and the isolated vessel chamber was constantly warmed to maintain a temperature of 38 ± 0.1 °C in the D-MEM/F12 surrounding the vessel. Imaging of MLVs was performed using an Olympus LUCPLFLN 20× objective (0.45 N.A.) and an Olympus DP72 camera controlled by Olympus CellSens digital imaging software. Initially, we captured images in control conditions; then, we replaced solution for perfusion with D-MEM/F12 containing fluorescently labeled killed *S. aureus* (Wood strain without protein A), Alexa Fluor 594, Molecular Probes, Inc., sold through Life Technologies. After 45 min after the bacterial perfusion begun, we closed inlet stopcock to prevent passive axial flow to see potential elimination of bacteria from MLVs segments. We continued observations of the MLVS segments up to 3 h continuously monitoring them with capturing of the images every 5 min. After the experiments, captured images were analyzed manually to compare axial and transmural distribution of bacteria in adult and aged MLVs.

### Sample preparation for FACS analysis and CFU

Following *C. neoformans* or *M. smegmatis* injection in the footpad, calves were harvested at different time points and dissociated and digested with collagenase (*Clostridium histolyticum*, Sigma) for 15 min at 37 °C. Single-cell suspension was filtered through a 100-μm Cell Strainer (Falcon). In some experiments, the digested samples were used for CFU determination for *C. neoformans* and *M. smegmatis* as described above. Colonies were normalized to tissue protein concentration as determined by Bradford. In other experiments, cells were incubated with saturating amount of the following antibodies: Alexa Fluor 488 rat anti-mouse CD11b (BD Biosciences, San Diego, CA, USA), Alexa Fluor 488 rat IgG2 isotype control, FITC hamster anti-mouse CD11c, and FITC hamster IgG1 isotype control (all from BD Biosciences) for 30 min on ice in staining buffer (PBS, 1% FCS, 0.01% NaN_3_). Following three washing in staining buffer, samples were analyzed by FACS using a flow cytometer LSR II (Becton Dickinson, San Jose, CA, USA).

### Rat mesenteric lymphatic vessels proteomics analysis

Mesenteric lymphatic vessels were collected from 9- and 24-month-old rats. The total proteins were extracted in lysis buffer (150 mm NaCl, 50 mm Tris–HCl, 1 mm EGDA, 1% Np40, pH 8.0) in the presence of protease inhibitors cocktail (La Roche, San Francisco, CA, USA), on ice for 30 min. The total lysates were further split in half, and one part was processed with the Thermo Scientific Pierce Glycoprotein Isolation Kit (Rockford, IL,USA) WGA to isolate glycoproteins, while the rest of lysates were used for global proteomic profiling.

Both total cell lysates and the eluted glycoprotein, from 9- and 24-month-old MLVs rats, were fractionated by 1D SDS–PAGE. The gels were silver-stained (Thermo Scientific). Eight gel bands were cut across each lane before the mass spectroscopy analysis. Reduction was performed for 30 min at room temperature with 10 mm TCEP.HCl (Thermo Scientific) in 50 mm ammonium bicarbonate buffer, pH 8.5. The in-gel reduced proteins were further alkylated with 55 mm iodoacetamide solution in 50 mm ammonium bicarbonate. Four different enzymes were used for the in-gel digestion: first bands were digested for 18 h, at 37 °C with endoproteinase Lys-C (sequencing grade, Promega, Madison, WI, USA) (1:50, protein: enzyme ratio) in 50 mm ammonium bicarbonate buffer, pH 8.5. Then, tryptic digestion was performed for 3 h at 37 °C, in 50 mm ammonium bicarbonate buffer, at pH 8.5 (1:50, protein: enzyme ratio). Finally, Asp-N or Glu-C were added (1:10 Asp-N or Glu-C: protein ratio) in ammonium 50 mm bicarbonate buffer (pH 7.5) at 37 °C for 10 h. The total peptides, extracted from all enzymatic digestions, were combined, desalted on C18 Prep clean columns, and further subjected to nanoLC–ESI–MS/MS on Orbitrap.

NanoLC/MS/MS with a Waters NanoAcquity HPLC system interfaced to a ThermoFisher Q Exactive was used to analyze the digested peptides. Operating in a data-dependent mode, the mass spectrometer with the Orbitrap operating at 60 000 FWHM and 17 500 FWHM for MS and MS/MS, respectively, was used. The fifteen most-abundant ions were selected for MS/MS. Raw data files were converted to mgf files using Proteome Discoverer 1.3 (Thermo Fisher Scientific, Rockford, IL,USA).

Proteome Discoverer (version 1.3; Thermo Scientific) was used to generate the mgf files from the raw data generated by the nanoLC Orbitrap system. All mgf files were analyzed using Mascot (Matrix Science, London, UK; version 2.4.1), with trypsin, Lys-C, Asp-N, and Glu-C restriction for enzymes and with a fragment ion mass tolerance of 0.80 Da and a parent ion tolerance of 10.0 PPM. The tandem MS/MS files were searched against the updated Swissprot_AC database containing all entries for *Mus musculus* (updated *June 2013*). A false discovery rate (FDR) for peptide identification was assessed by decoy database searching and was finally adjusted to less than 5.0% for proteins and less than 2% for peptides. The following posttranslational modifications were specified in Mascot as variable modifications: mono-oxidation of arginine, lysine, methionine and proline (+15.99), proline conversion to pyrrolidone (−28.00), proline conversion to pyrrolidinone (−30.00), conversion of lysine to lysine-HNE (+156.12), conversion of lysine to carboxymethyl lysine (+58), and to carboxyethyl lysine, conversion of arginine to gamma-glutamyl-semialdehyde (GluSA) (−43), and conversion of arginine to 3-deoxyglucosone (+144.14). The proteins were considered identified having at least one bold red significant peptide with an ion score cutoff of 28 or greater (corresponding to *P* < 0.05 and a FDR protein <1.0).

Scaffold (version Scaffold_4.0.7, Proteome Software Inc.) was used to validate MS/MS-based peptide and protein identifications. Peptide identifications were accepted if they could be established at greater than 95.0% probability by the Peptide Prophet algorithm with Scaffold delta-mass correction. Protein identifications were accepted if they could be established at greater than 90.0% probability and contained at least 1 identified peptide. Protein probabilities were assigned by the Protein Prophet algorithm. Proteins that contained similar peptides and could not be differentiated based on MS/MS analysis alone were grouped to satisfy the principles of parsimony. Proteins were annotated with GO terms from NCBI (downloaded on November 1, 2013).

### Generation of heat maps

The relative protein levels were estimated by calculating the normalized spectral abundance factors (NSAF) as described (Zybailov *et al*., 2006; Sardiu & Washburn, 2010). The three independent biological replicates for each sample group were combined into a MudPIT (multidimensional protein identification technology) using the Scaffold 4.0 built-in option. The natural logarithm transformation of the NSAF [Ln(NSAF)] was used to perform a relative label-free quantification of the protein expression in each biological sample. The Ln(NSAF) were used to generate the unclustered heat map for the two different sample sets (9 and 24 months). The heat maps were generated using the ArrayTrack™ (http://www.fda.gov/ScienceResearch/BioinformaticsTools/Arraytrack/).

### Permeability assay

Human dermal lymphatic endothelial cells were isolated from neonatal human foreskins and characterized as previously described (Hirakawa *et al*., 2003). Cells were cultured in endothelial basal medium (EBM; Cambrex) supplemented with 20% fetal bovine serum (FBS) (GIBCO), antibiotic antimycotic solution (1×; Fluka), L-glutamine (2 mm; Fluka, St. Louis, MO, USA), hydrocortisone (10 μg mL^−1^; Fluka), and N^6^, 2′-O-dibutyryladenosine 3′,5′-cyclic monophosphate sodium salt (cAMP, 2.5 × 10^−2^ mg mL^−1^; Fluka) and grown at 37 °C in 5% CO_2_ for up to ten passages.

For the permeability assay, 100 000 LECs were seeded in 150 μL of medium into the fibronectin-coated upper chamber of Transwell plates (0.4 μm pore size, 6.5 mm diameter, clear polyester membrane, Corning Inc., Corning, NY, USA). The lower wells were filled with 700 μL of medium. Cells were grown to confluence for 2 days, and some wells (*n* = 3–4 wells/per condition) were subsequently treated overnight with paraquat (20 μm, Sigma-Aldrich). The next day, 50 μL of a 2.5 mg mL^−1^ FITC–dextran solution (70 kDa, Sigma-Aldrich) was added to the upper well and mixed by pipetting. After incubating for 15 min at 37 °C, 50 μL samples were collected from the lower chambers and transferred to a clear-bottom, black-walled 96-well plate (Corning Inc.). Fluorescence at 520 nm was measured on a SpectraMAX Gemini EM (Molecular Devices, Bucher Biotech, Sunnyvale, CA, USA). As a positive control, some LEC monolayers were treated with VEGF-A (Peprotech) 20 min prior to the addition of FITC–dextran. Results are shown as data subtracted by the background media control.

### Evans blue injections and measurement of collector’s leakage

Footpads of the lower limbs of 4- and 22-month-old mice were injected with 30 μL of 2–5% Evans blue solution in PBS. After 20 min, mice were anesthetized and perfused *via* the left ventricle with PBS. Collectors in the calf region were visualized at the stereomicroscope and imaged using an Olympus LUCPLFLN 20× objective (0.45 N.A.)

## Supporting Information

**Fig. S1**Compromised pathogens transport and increased lymphatic permeability in aging mice. **Table S1** Proteomic Analysis of Lymphatic Collectors harvested from 9 and 24 months old rats.

**Movie S1a** Contractile activity of rat mesenteric lymphatic collector (9 months old).

**Movie S1b** Contractile activity of rat mesenteric lymphatic collector (24 months old).

**Movie S2a**: Bacterial transport in a 4 months old mouse limb lymphatic collector (GFP-S. Aureus; TRITC-Dextran).

**Movie S2b**: Bacterial transport in a 22 months old mouse limb lymphatic collector (GFP-S. Aureus; TRITC-Dextran).

**Movie S2c**: Bacterial transport in a 22 months old mouse limb lymphatic collector (GFP-S. Aureus; TRITC-Dextran).

**Movie S3**: Bacterial transport in a 4 months old mouse limb lymphatic collector (GFP-S. Aureus; PEG-D680).
