# Supplementary material for: Aging-related anatomical and biochemical changes in lymphatic collectors impair lymph transport, fluid homeostasis, and pathogen clearance
Source: Aging Cell. 2015 May 15;14(4):582–94. doi: 10.1111/acel.12330 (PMC4531072; doi:10.1111/acel.12330)
Supplement: Supplementary file 1 [file acel0014-0582-sd1.pdf]

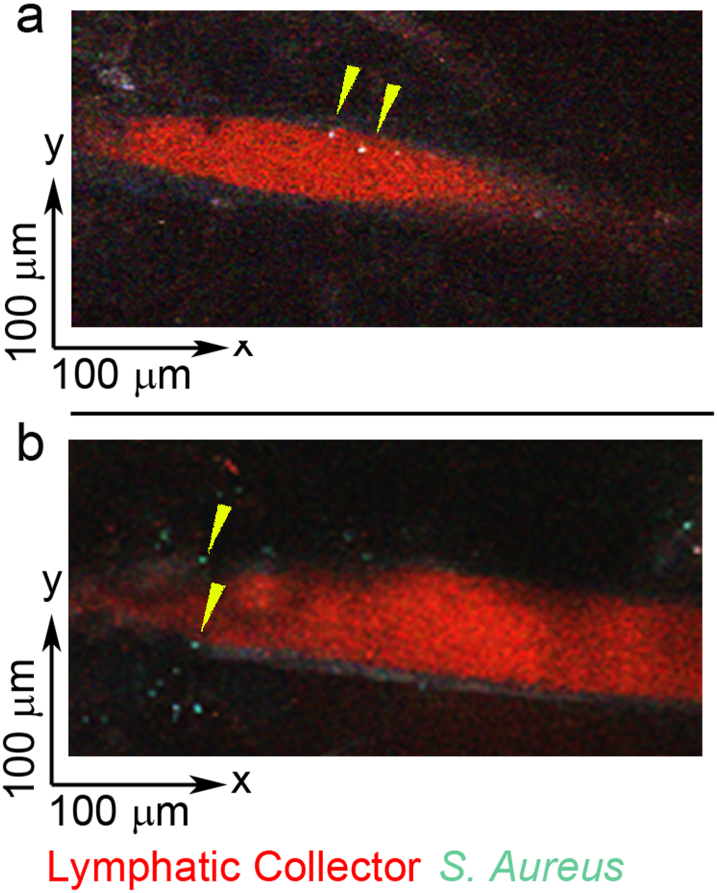

Figure S1 Compromised pathogens transport and increased lymphatic permeability in aging mice.

Representative (xy and yz) images of lymphatic collectors in the hind leg taken 1 hour after footpad injection with a TRITC-dextran sinus marker (red) and *Staphilococcus. Aureus* (cyan) bacteria in the footpad from 4 month (f) and 23 month-old mice (g). Bacteria in vessels highlighted with yellow arrows.

Supplement Table S1
Proteomic Analysis of Lymphatic Collectors
harvested from 9 and 24 months old rats

Proteins	Accession Number
Collagen alpha-1(I) chain	CO1A1_RAT
Collagen alpha-1(II) chain	CO2A1_RAT
Collagen alpha-1(III) chain	CO3A1_RAT
Collagen alpha-1(V) chain	CO5A1_RAT
Collagen alpha-1(XI) chain	COBA1_RAT
Collagen alpha-1(XII) chain (Fragment)	COCA1_RAT
Collagen alpha-1(XXIII) chain	CONA1_RAT
Collagen alpha-1(XXVII) chain	CORA1_RAT
Collagen alpha-2(I) chain	CO1A2_RAT
Fibronectin	FINC_RAT
Fibronectin type III domain-containing protein 1	FNDC1_RAT
Fibronectin type-III domain C1orf233 homolog	CA233_RAT
Cartilage oligomeric matrix protein	COMP_RAT
Extracellular matrix protein 1	ECM1_RAT
Non-muscle caldesmon	CALD1_RAT
Prelamin-A/C	LMNA_RAT
Alpha-actinin-1	ACTN1_RAT
Gelsolin	GELS_RAT
Dynactin subunit 1	DCTN1_RAT
Dynein assembly factor 1, axonemal	DAAF1_RAT
Dynein heavy chain 1, axonemal	DYH1_RAT
Dynein heavy chain 7, axonemal	DYH7_RAT

**Troponin I, slow skeletal muscle**	TNNI1_RAT
**Troponin T, slow skeletal muscle**	TNNT1_RAT
**Unconventional myosin-Ib**	MYO1B_RAT
**Unconventional myosin-Ic**	MYO1C_RAT
**Unconventional myosin-Id**	MYO1D_RAT
**Myosin light chain 1/3, skeletal muscle isoform**	MYL1_RAT
**Myosin light chain 3**	MYL3_RAT
**Myosin light polypeptide 6**	MYL6_RAT
**Myosin-10**	MYH10_RAT
**Myosin-4**	MYH4_RAT
**Myosin-6**	MYH6_RAT
**Myosin-8 (Fragment)**	MYH8_RAT
**Myosin-9**	MYH9_RAT
**Myosin-binding protein H-like**	MBPHL_RAT
**Actin filament-associated protein 1**	AFAP1_RAT
**Actin, cardiac muscle 1**	ACTC_RAT
**Actin-like protein 7A**	ACL7A_RAT
**Actin-related protein 2/3 complex subunit 1A /5**	ARC1A_RAT
**Actin-related protein 3**	ARP3_RAT
**Afadin- and alpha-actinin-binding protein**	ADIP_RAT
**Afadin**	AFAD_RAT
**Myogenin**	MYOG_RAT
**Myomegalin**	MYOME_RAT
**Myotubularin**	MTM1_RAT
**Myotubularin-related protein 3**	MTMR3_RAT
**Angiomotin-like protein 2**	AMOL2_RAT
**Voltage-dependent T-type calcium channel subunit alpha-1G**	CAC1G_RAT
**Voltage-dependent L-type calcium channel subunit alpha-1C**	CAC1C_RAT
**Plasma membrane calcium-transporting ATPase 3**	AT2B3_RAT
**Plasma membrane calcium-transporting ATPase 1**	AT2B1_RAT
**Potassium channel subfamily K member 10**	KCNKA_RAT
**Potassium voltage-gated channel subfamily A member 6**	KCNA6_RAT

**Potassium voltage-gated channel subfamily G member 2**	KCNG2_RAT
**Potassium voltage-gated channel subfamily KQT member 4**	KCNQ4_RAT
**Potassium-transporting ATPase alpha chain 2**	AT12A_RAT
**Sodium channel protein type 4 subunit alpha**	SCN4A_RAT
**Sodium channel protein type 5 subunit alpha**	SCN5A_RAT
**Sodium channel protein type 9 subunit alpha**	SCN9A_RAT
**Calcium-activated potassium channel subunit alpha-1**	KCMA1_RAT
**Alpha-1B adrenergic receptor**	ADA1B_RAT
**Alpha-2A adrenergic receptor**	ADA2A_RAT
**Beta-adrenergic receptor kinase 1**	ARBK1_RAT
**Agrin**	AGRIN_RAT
**Endothelial PAS domain-containing protein 1**	EPAS1_RAT
**Clusterin**	CLUS_RAT
**Clusterin-like protein 1**	CLUL1_RAT
**Hyaluronan and proteoglycan link protein 2**	HPLN2_RAT
**Hyaluronan synthase 2**	HAS2_RAT
**Ig-like V-type domain-containing protein FAM187A**	F187A_RAT
**Aggrecan core protein**	PGCA_RAT
**Versican core protein (Fragments)**	CSPG2_RAT
**Chondroitin sulfate proteoglycan 5**	CSPG5_RAT
**Chondroitin sulfate proteoglycan 4**	CSPG4_RAT
**Brevican core protein**	PGCB_RAT
**Alpha-2-HS-glycoprotein**	FETUA_RAT
**Versican core protein (Fragments)**	CSPG2_RAT
**Neurocan core protein**	NCAN_RAT
**Galectin-4**	LEG4_RAT
**Mucin-2 (Fragment)**	MUC2_RAT
**Amiloride-sensitive amine oxidase [copper-containing]**	AOC1_RAT
**Amiloride-sensitive sodium channel subunit alpha**	SCNNA_RAT

**Anion exchange protein 2**	B3A2_RAT
**Anion exchange protein 3**	B3A3_RAT
**Cubilin**	CUBN_RAT
**Band 3 anion transport protein**	B3AT_RAT
**Chloride channel protein 2**	CLCN2_RAT
**Chloride anion exchanger**	S26A3_RAT
**Chloride channel protein 1**	CLCN1_RAT
**Chloride intracellular channel protein 6**	CLIC6_RAT
**Sodium/calcium exchanger 1**	NAC1_RAT
**Sodium/hydrogen exchanger 2**	SL9A2_RAT
**Sodium/hydrogen exchanger 5**	SL9A5_RAT
**Sodium-dependent phosphate transport protein 2B**	NPT2B_RAT
**Sodium-driven chloride bicarbonate exchanger**	S4A10_RAT
**Transient receptor potential cation channel subfamily M member 1**	TRPM1_RAT
**Electrogenic sodium bicarbonate cotransporter 1**	S4A4_RAT
**Electrogenic sodium bicarbonate cotransporter 4**	S4A5_RAT
**Nephrin**	NPHN_RAT
**Junction plakoglobin**	PLAK_RAT
**Basal cell adhesion molecule**	BCAM_RAT
**Cadherin EGF LAG seven-pass G-type receptor 2 (Fragment)**	CELR2_RAT
**Cadherin EGF LAG seven-pass G-type receptor 3**	CELR3_RAT
**Cadherin-1**	CADH1_RAT
**Cadherin-17**	CAD17_RAT
**Cadherin-2**	CADH2_RAT
**Cadherin-20**	CAD20_RAT
**Cadherin-23**	CAD23_RAT
**Cadherin-6**	CADH6_RAT
**Cadherin-7**	CADH7_RAT
**Integrin alpha-7**	ITA7_RAT
**Integrin alpha-D**	ITAD_RAT
**Integrin-linked kinase-associated serine/threonine phosphatase 2C**	ILKAP_RAT
**Integrin-linked protein kinase**	ILK_RAT

**Contactin-4**	CNTN4_RAT
**Desmoglein-4**	DSG4_RAT
**Gap junction gamma-2 protein**	CXG2_RAT
**Gap junction alpha-4 protein**	CXA4_RAT
**Protocadherin alpha-4**	PCDA4_RAT
**Protocadherin Fat 2**	FAT2_RAT
**Protocadherin Fat 3**	FAT3_RAT

Lymphatic Muscle Contraction
Extracellular Matrix Proteins
Adrenergic Receptors
Membrane Potential-related Ion Channel Proteins
Glycocalix-associated Proteins
Ion Channel-related proteins
Adherence Molecules
